# Malignancy rates of B3-lesions in breast magnetic resonance imaging – do all lesions have to be excised?

**DOI:** 10.1186/s12880-018-0271-7

**Published:** 2018-09-10

**Authors:** H. Preibsch, L. K. Wanner, A. Staebler, M. Hahn, K. C. Siegmann-Luz

**Affiliations:** 1 Department of Diagnostic and Interventional Radiology, University Hospital Tuebingen, Hoppe-Seyler-Str. 3, 72076 Tuebingen, Germany; 2 Department of Pathology and Neuropathology, University Hospital Tuebingen, Liebermeisterstr. 8, 72076 Tuebingen, Germany; 3 Department of Obstetrics and Gynecology, University Hospital Tuebingen, Calwerstr. 7, 72076 Tuebingen, Germany; 4 Diagnostic Breast Centre and Breast Cancer Screening Brandenburg East, Koepenicker Str. 29, 15711 Koenigs Wusterhausen, Germany

**Keywords:** B3 lesions, Vacuum-assisted biopsy, Breast MRI, MRI-guided breast biopsy

## Abstract

**Background:**

Approximately 10% of all MRI-guided vacuum-assisted breast biopsies (MR-VAB) are histologically classified as B3 lesions. In most of these cases surgical excision is recommended. The aim of our study was to evaluate the malignancy rates of different B3 lesions which are visible on MRI to allow a lesion-adapted recommendation of further procedure.

**Methods:**

Retrospective analysis of 572 consecutive MR-VAB was performed. Inclusion criteria were a representative (=successful) MR-VAB, histologic diagnosis of a B3 lesion and either the existence of a definite histology after surgical excision or proof of stability or regression of the lesion on follow-up MRI. Malignancy rates were evaluated for different histologies of B3 lesions. Lesion size and lesion morphology (mass/non-mass enhancement) on MRI were correlated with malignancy.

**Results:**

Of all MR-VAB 43 lesions fulfilled the inclusion criteria. The malignancy rate of those B3 lesions was 23.3% (10/43). The highest malignancy rate was found in atypical ductal hyperplasia (ADH) lesions (50.0%; 4/8), 33.3% (2/6) in flat epithelial atypia (FEA), 28.6% (2/7) in lobular intraepithelial neoplasia (LIN) and 12.5% (2/16) in papillary lesions (PL). All 6 complex sclerosing lesions were benign. Mass findings were significantly more frequently malignant (31.3%, 10/32; *p* < 0.05) than non-mass findings (0/11). Small lesions measuring 5–10 mm were most often malignant (35.0%; 7/20). All large lesions (> 20 mm) were not malignant (0/10). Intermediate sized lesions (11–20 mm) turned out to be malignant in 23.1% (3/13).

**Conclusions:**

The malignancy rate of B3 lesions which were diagnosed after MR-VAB was 23.3%. ADH, FEA and LIN showed considerable malignancy rates (50%, 33% and 29%) and should therefore undergo surgical excision. None of the cases, which were diagnosed as radial scars, non-mass enhancement or larger lesions (> 20 mm) were malignant. Here, a follow-up MRI seems to be advisable to avoid unnecessary operations.

**Trial registration:**

Retrospective study design, waived by the IRB.

## Background

Magnetic resonance imaging (MRI) of the breast is the most sensitive breast imaging modality. It is especially useful in patients with newly diagnosed breast cancer and high breast density on mammography, recurrent breast cancer and screening of high-risk patients. The increasing use of breast MRI results in an increasing number of suspicious findings categorised as category 4 or category 5 according to the Breast Imaging Reporting and Data System (BI-RADS®) [1]. Those lesions demand histologic clarification. If no correlating lesion can be found on second-look breast ultrasound or mammography, MRI-guided vacuum-assisted biopsy (MR-VAB) is necessary. Biopsy specimens of the breast are classified into five histologic categories according to current european guidelines [2] which are based on the international WHO classification of tumours of the breast. Breast lesions which are benign but have an uncertain malignant potential are categorised as B3. This heterogenous group of different histologies is accompanied by an increased risk of associated malignancy. The rate of malignancy in surgical excision of mammographic B3 lesions varies between 0 and 30%, with the highest malignancy rate in ADH (20–30%) [3, 4]. Therefore a surgical biopsy is mostly recommended [5–7]. Approximately 10% of all MRI-BI-RADS® 4 and BI-RADS® 5 lesions clarified by MR-VAB turn out to be B3 lesions [8, 9]. To reduce the rate of benign surgical biopsies of MRI detected B3 lesions it would be desirable to identify low-risk and high-risk lesions before surgery. Until now there is very limited data about the malignancy rate of MRI-only B3 lesions. Therefore the clinical management of these cases is basically derived from the management of mammographic B3 lesions. Hence, the decision on the clinical management of MRI detected B3 lesions is made intuitively in each individual case and varies between different institutions.

The aim of our study was to evaluate the malignancy rate of different MRI-detected and biopsy-proven B3 lesions to optimise clinical management of these lesions due to a reduction of benign surgical excisions. Furthermore we wanded to evaluate the influence of different factors (lesion size, lesion type, patient age, history of breast cancer) on the malignancy rate to derive a management algorithm for clinical routine.

## Methods

### Patiens and lesions

We retrospectively reviewed the data of 572 consecutive MR-VAB which were carried out during a 100-month period at our institution. Written informed consent was waived by the Institutional Review Board (No. 372/2017BO2). Inclusion criteria were a histologically diagnosed B3 lesion by MR-VAB and either performed excisional biopsy or follow-up breast MRI at our institution. The success of MRI-guided biopsy had to be confirmed by a complete or partial lesion removal on dynamic contrast enhanced breast MRI short term after intervention, usually on the next day. Further MRI follow-up data over a time period of at least 1.5 years had to be available if the targeted B3-lesion was not completely removed or if it did not decrease in size on first follow-up MRI.

Altogether 9.4% (54/572) of the reviewed MRI-guided biopsies revealed a B3 lesion. Four of them had to be excluded because excisional biopsy or follow-up breast MRI was carried out in an external institution or not done at all. Five other patients were lost on follow-up MRI. In 2 patients, the histology of excisional biopsy could not be correlated with the previously diagnosed B3 lesion because the lesion localisation was unclear and no marker clip was set after MR-VAB.

Hence, 43 patients with 43 lesions met the inclusion criteria and could be taken for final analysis. The majority of patients underwent breast MRI due to newly diagnosed breast cancer (28/43; 65.1%). Ten of them presented with ipsilateral breast cancer, 17 patients had contralateral breast cancer and one patient had bilateral breast cancer. Five patients were followed-up after excisional biopsy (5/43; 11.6%) and another five patients had equivocal findings on mammography and ultrasound (5/43; 11.6%). Three patients had a history of breast cancer of the contralateral breast (3/43; 7.0%). In 4.7% (2/43) breast MRI was performed for the clarification of clinical symptoms (*n* = 1 nipple retraction, *n* = 1 nipple discharge).

Mean patient age was 52.0 years (30–81 years). All of them received a diagnostic contrast enhanced breast MRI 2 to 53 days (mean 12 days) before MRI-guided biopsy. Second-look breast ultrasound was performed in all cases without proof of any correlating lesion. The biopsied lesions were visible on MRI only, so MR-guided biopsy was indicated. Thirty-four of them were categorised as BI-RADS® 4 (79.5%) and 9 were categorised as BI-RADS® 5 (20.5%) on MRI.

### MRI protocols

Breast MR Imaging in the diagnostic as well as in the follow-up setting was performed at 1.5 T (Achieva, Philips Healthcare, Amsterdam, Netherlands) with a dedicated double breast coil (SENSE breast coil, Philips Healthcare, Amsterdam, Netherlands). After a T2 weighted (T2w) short tau inversion recovery (STIR) sequence in transversal plane (repetition time, 3200 ms; echo time, 50 ms; inversion time, 160 ms; matrix, 512 × 512 pixels; field of view, 360 mm; slice thickness, 3.5 mm), T1w gradient echo sequences (repetition time, 7.5 ms; echo time, 3.7 ms; matrix, 512 × 512 pixels; field of view, 400 mm; flip angle, 20°; slice thickness, 1.5 mm) were acquired before and after i.v. injection of gadolinium contrast agent (0.1 mmol/kg body weight Gadobutrol, Gadovist®, Bayer HealthCare AG, Berlin, Germany). The dynamic series consisted of one unenhanced and seven series after contrast agent injection. Subtraction images of each series were calculated.

MR-guided VAB was performed using the 1.5 T MR machine described above using a dedicated breast surface coil. No T2w sequence was acquired, but a T1w dynamic series in the transversal plane (parameters and contrast agent: see above). Unenhanced, non fat-suppressed T1w sequences in the transveral plane were used as control imaging and an additional sagittal plane after the clip placement. Two different vacuum-assisted breast biopsy systems were used (11G Mammotome®, Devicor, Medical Products, Cincinnati, OH, USA; 10G Vacora®, BARD Biopsy Systems, Karlsruhe, Germany). Partial or total removal of the suspicious lesion was confirmed by a short-interval follow-up MRI one or two days after the MR-guided intervention.

### Excisional biopsy and follow-up MRI

In the majority of B3 lesions (33/43; 76.7%) a surgical excision was performed. Thereby the lesions were either removed by a single excisional biopsy (20/33; 60.6%) or during breast conserving therapy or mastectomy because of synchronous ipsilateral breast cancer (13/33 = 39.4%). In 10 patients (23.3%) no surgical resection but MRI follow-up was performed. Reasons for follow-up instead of surgical excision were: a complete lesion removal on short-interval follow-up MRI, a very small lesion which was verifiable histologically only in one biopsy specimen, and the complete disappearance of the lesion on follow-up MRI after the start of neoadjuvant chemotherapy of a contralateral breast cancer. The time interval between MR-VAB and latest MRI follow-up was mean 26.5 months (range 6 to 66 months). The last control MRI showed a disappearance of the B3 lesion in 6 cases (60%), a lesion which was stable in size and contrast enhancement in 2 cases (20%), and a decreased lesion size in 2 cases (20%). In 2 lesions control intervals were shorter than 1.5 years – and showed either disappearance or a decreasing size of the lesion during MRI follow-up. Therefore all lesions with MRI follow-up (*n* = 10) were classified as benign.

### Histopathology – Categories of B3 lesions

All histopathologic diagnoses were made by experienced breast pathologists according to current guidelines [2]. The diagnosed B3 lesions were categorised in 5 groups: Papillary lesions (PL) with or without epithelial atypia, flat epithelial atypia (FEA), radial scar respectively complex sclerosing lesions with or without epithelial atypia (RS), lobular intraepithelial neoplasia (LIN), atypical ductal hyperplasia (ADH). Lobular intraepithelial neoplasia lesions were subdivided into LIN 1, LIN 1–2 and LIN 2. There were no LIN 3 findings because they are considered as malignant lesions and therefore classified as B5a.

### Data analysis

All hospital related data like medical history, clinical management, surgery, histopathology and imaging findings were taken from the Hospital Information System (KIS) and the Radiology Information System (RIS) of our institution.

Breast MR images were reviewed by two radiologists in consensus who had one and 14 years experience in reading breast MRI (L. K. W., K. C. S.-L.). MR images were analysed using a dedicated workstation (EWS, Philips Healthcare, Hamburg, Germany). Contrast kinetics were analysed by manually drawn regions of interest (ROI) on the subtraction images.

**Fig. 1:**
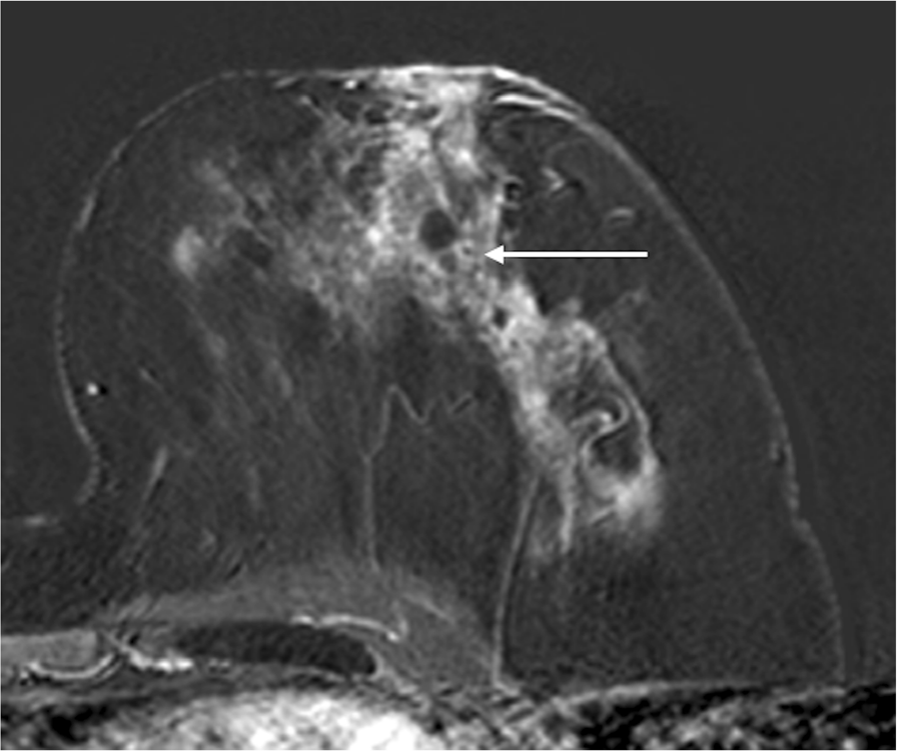
Non-mass enhancement of 90 mm (subtraction images) in the upper outer quadrant of the left breast. MR-guided VAB (in 40 mm nipple distance, arrow) revealed benign histology (radial scar)

**Fig. 2:**
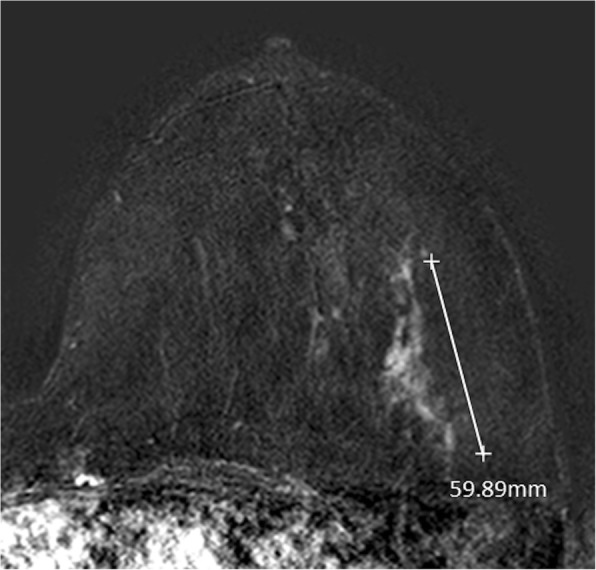
Non-mass enhencement measuring 60 mm (subtraction images) in the lower outer quadrant of the left breast. MR-VAB had benign histology (papillary lesions and FEA) as a result

**Fig. 3:**
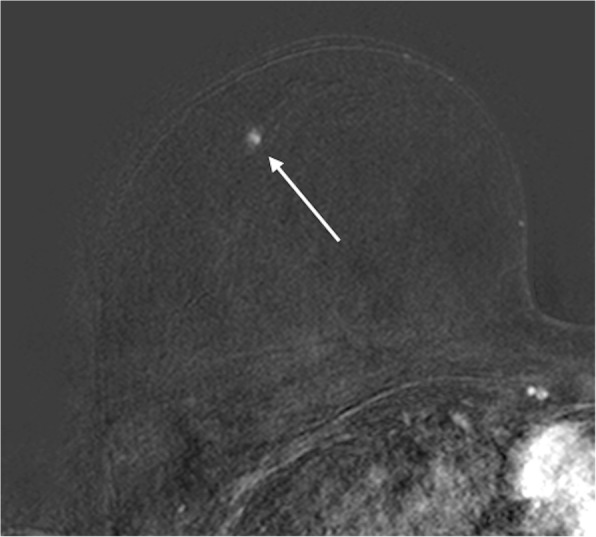
Subtraction images with contrast-enhancing mass lesion of 7 mm (arrow) in the upper outer quadrant of the right breast, which showed atypical epithelial proliferation of ductal type (B3) on MR-guided biopsy, but proved to be invasive ductal carcinoma (grade 2) on final histology

**Fig. 4:**
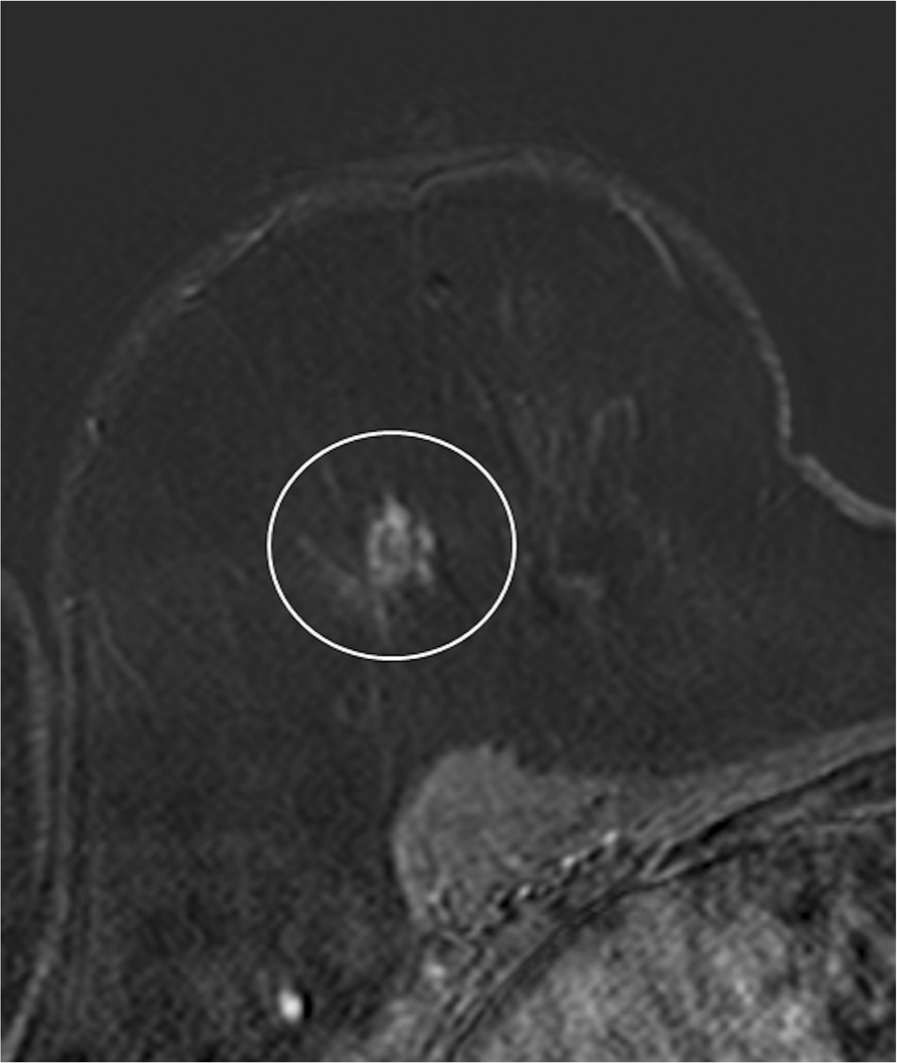
Irregular shaped mass lesion (subtraction images) in the upper outer quadrant of the right breast with a size of 13 mm. MR-guided VAB showed papillary lesion and ADH on histopathology, but final histology confirmed low grade DCIS

**Fig. 5:**
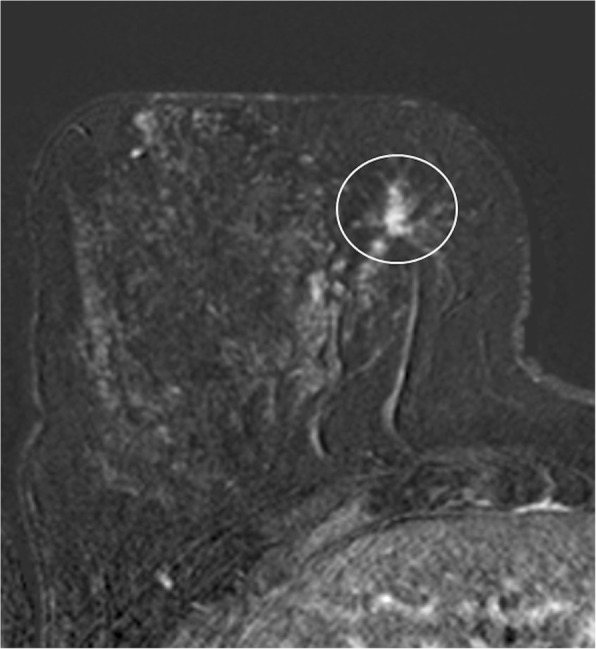
MR-guided VAB of this 15 mm measuring mass lesion (subtraction images) in the lower inner quadrant of the right breast showed benign histology (radial scar in association with lobular intraepithelial neoplasia)

**Fig. 6:**
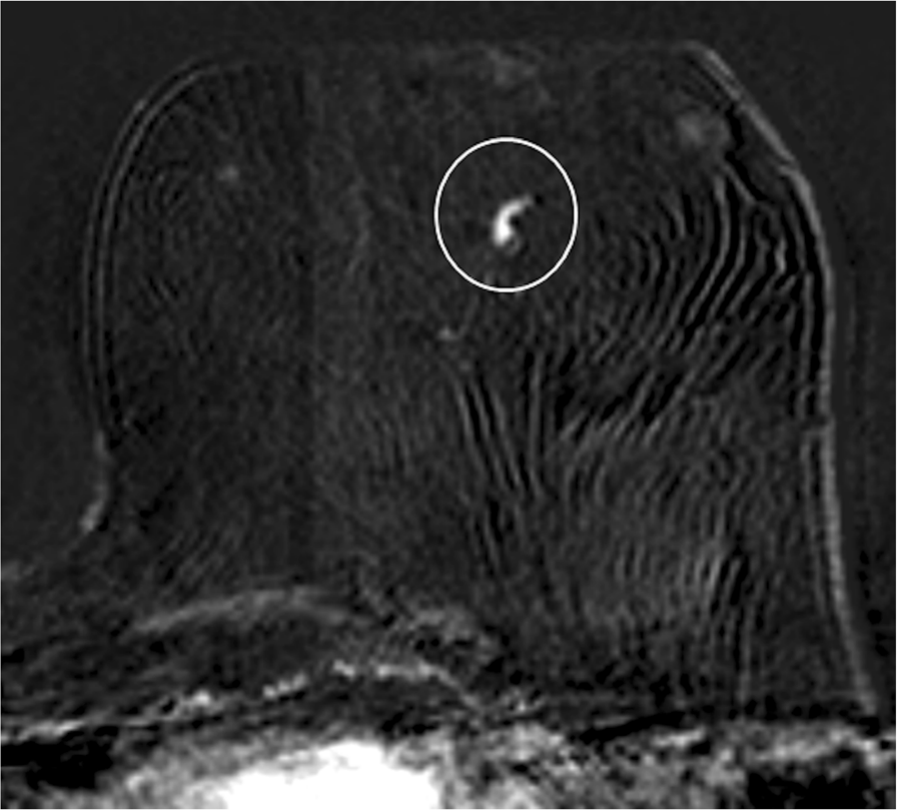
This 8 mm measuring mass lesion (subtraction images) in the center of the left breast had benign histology (radial scar)

According to MRI-BI-RADS and lesion morphology all lesions were classified either as non-mass (*n* = 11; 25.6%; see Figs. 1 and 2) or mass lesions (*n* = 32; 72.7% see Figs. 3, 4, 5 and 6). The lesion size was measured manually by means of a dedicated workstation and categorised corresponding to the TNM-classification: ≤5 mm: *n* = 0; > 5 mm and ≤ 10 mm: *n* = 20; > 10 and ≤ 20 mm: *n* = 13; > 20 mm: *n* = 10. Due to the small numbers large lesions of more than 50 mm were not seperately analysed.

The malignancy rate of MRI-only B3 lesions was evaluated. To evaluate the influence of histopathologic type, MRI lesion type, MRI lesion size, and patients’ history of breast cancer on the malignancy rate a correlation analysis of those variables with histology (malignant or benign) was performed.

### Statistics

Statistical analysis was performed using the chi-squared test to test for stochastic independency. Correlation of the different variables (patient age, risk anamnesis, lesion size, lesion morphology and histology) with the malignancy rate was determined by using a cross table with calculation of 95% confidence intervals. Significant correlation was considered in case of *p* ≤ 0.05. Analysis was made using statistical programmes (SPSS 16.0; SPSS, Chicago, IL; JMP 12, SAS, Cary, NC).

## Results

### Malignancy rate of different B3 lesions

The malignancy rates of each subgroup of B3 histologies are shown in Tables 1 and 2. Papillary lesions were the most frequent B3 lesions (16/43; 37.2%). Two of those lesions (12.5%) turned out to be malignant in excisional biopsy (DIN 1c and DIN 2). Four of the benign lesions were only followed up (see column “no excision histology”, Table 1) and appeared constant or disappeared after at least 1.5 years. The final histology of surgical excision was a papillary lesion (*n* = 5), a papillary lesion in combination with FEA (*n* = 2), ADH (*n* = 1), LIN 1 (*n* = 1) and only normal breast parenchyma (B1, *n* = 1) due to complete removal of the B3 lesion through MR-VAB.

**Table 1:** Outcome stratified into the different histologic entities classified as B3

Type of B3 lesion	Final histology					
	Benign	DCIS	Malignant invasive	No excision histology	PPV (%)	Total
PL	10	2	0	4	13	16
Radial scar	4	0	0	2	0	6
FEA	3	0	2	1	33	6
*LIN 1*	1	0	0	1	0	2
*LIN 1–2*	0	1	0	0	100	1
*LIN 2*	3	0	1	0	25	4
LIN total	4	1	1	1	29	7
ADH	2	1	3	2	50	8
Total	23	4	6	10	24	43

Papillary lesion (PL); LIN lesions are listed subdivided into LIN 1, LIN 1–2 and LIN 2 lesions (italic), and in total

**Table 2:** Outcome dependent on individual risk anamnesis of the patients

Risk anamnesis			
	Total	Malignant	% Malignant
Proven ipsilateral Ca	10	5	50
Proven contralateral Ca	17	3	18
Proven bilateral Ca	1	0	0
No present or history of Ca (partly familiar high risk situation)	9	1	11
Mamillary retraction/ secretion	2	0	0
History of Ca	4	1	25

Average age of patients with PL was 52.2 years.

Six of the 43 B3 lesions (14.0%) were classified as radial scar complexes. All 6 turned out to be benign: two of the lesions were controlled by follow-up MRI, both decreasing in size after 9 months or more than 3 years, respectively. Mean patient age in this group was 48.7 years.

In 6 patients (14.0%) a FEA could be identified in MR-VAB. Two of the lesions turned out to be invasive carcinoma in excisional biopsy (33.3%). The benign lesions were associated with another B3 lesion in excisional biopsy in two cases (papillary lesion and radial scar complex, respectively). Mean age of patients with this entity was 50.5 years.

LIN was identified in 7 patients. Here, two lesions (28.6%) turned out to be malignant in final histology: one invasive carcinoma and one DIN 2 lesion. More detailed, the two malignant lesions were reported as LIN 1–2 and LIN 2 in MR-VAB, the benign lesions were LIN 1 (*n* = 2) and LIN 2 (*n* = 3) (see Table 1). Patients with LIN lesions had a mean age of 57.1 years.

Eight of the 43 B3 lesions (18.6%) were classified as ADH, of which 4 lesions turned out to be malignant (50%). Three lesions turned out to be invasive carcinomas, one was classified as DIN 1c. Mean age of patients with ADH was 50.5 years.

The respective positive predictive values (PPV) are 50% for ADH, 33% for FEA, 29% for LIN, 13% for papillary lesions and 0% for radial scars. In total, the PPV for B3 lesions was 24% in our study (also shown in Table 1).

### Risk anamnesis and age of patient

Considering risk anamnesis of the patients, in 5 of 10 women (50%) with recently diagnosed carcinoma of the ipsilateral breast, the suspicious B3 lesion turned out to be malignant. In case of contralateral breast cancer, only 17.6% (3/17) of the B3 lesions had a malignant outcome (Table 2). In 25% of the patients with a history of breast cancer and in 11% of the patients without history of breast cancer the outcome was malignant. Risk anamnesis was a factor significantly influencing the malignancy rate (*p* = 0.016).

Considering the age of the patients worst outcome was detected in patients from 46 to 50 years: 6 out of 14 B3 lesions were malignant (42.9%). In the groups from 51 to 55 and older than 55 years, 20% of the lesions had a malignant final histology (2/10 each). Lesions in patients ≤45 years were in no case malignant (0/9) (see Table 3).

**Table 3:** Outcome dependent on patient age

Age				
	≤ 45	46–50	51–55	> 55
n	9	14	10	10
Benign	9	8	8	8
Malignant	0	6	2	2
% Malignant	0	43	20	20

Age range of patients with malignant final histology was 46 to 77 years (mean 53.4 years). Patients with benign outcome had a mean age of 52.0 years (30 to 81 years). Patient age was no significant factor influencing the malignancy rate acoording to cross tabulation (*p* = 0.530).

### Lesion size and morphology

B3 lesions measured between 5 and 90 mm (mean 18.1 mm) in largest diameter. Non-mass lesions were significantly greater than mass lesions with a mean size of 40.3 mm (7–90 mm) and 10.5 mm (5–24 mm), respectively. Seven out of 20 B3 lesions ≤10 mm had a malignant final histology (35.0%). Lesions from 11 to 20 mm were malignant in only 23.1% (3/13). Lesions taller than 20 mm were in none of the cases malignant (0/10) (see Table 4). Hence, lesions > 20 mm were significantly less likely to be malignant (*p* = 0.045).

**Table 4:** Outcome dependent on lesion size

Lesion size	≤ 10 mm	11–20 mm	> 20 mm
n	20	13	10
Benign	13	10	10
Malignant	7	3	0
% Malignant	35	23	0

Thirteen of the 16 papillary lesions (81.3%) had a mass aspect (including the two malignant lesions) and three (18.7%) were classified as non-mass lesions. Two of the radial scar complexes presented as mass (33.3%), 4 (66.7%) as non-mass lesions. Four of the 6 FEA lesions (66.7%) presented as masses on MRI, including both malignant lesions. All LIN lesions had mass appearance. Six of the 8 ADH lesions (75%) had mass appearance (including all 4 malignant lesions).

Hence, all malignant lesions had a mass aspect on diagnostic MRI (10/10). Non-mass lesions were benign in all cases (*p* = 0.031). Out of 33 lesions with benign outcome (final histology or follow-up MRI), 22 (66.7%) had mass morphology (see Table 5).

**Table 5:** Outcome dependent on lesion morphology

Lesion morphology	non-mass	mass	% masses
n	11	32	74
Benign	11	22	67
Malignant	0	10	100
% Malignant	0	31	

## Discussion

Some B3 lesions have the potential of malignant degeneration in terms of precancerous lesions, such as ADH [5] and possibly papillary lesions. Some B3 lesions are so-called “indicator lesions”, which are frequently associated with coexistent higher grade transformations, i.e. lobular neoplasia [6]. Analysing actual management of these lesions, practice is varying greatly among surgeons [6]. Because of several surveys reporting high rates of malignancy, routine excision is often recommended [5, 6]. More recent publications suggest vacuum-assisted excision instead of surgical excision in several B3 lesions [10].

As with the term B3 lesions a heterogenous group of lesions with different potential of malignany is summarised, some authors suggest a subclassification according to the presence of atypia into B3a and B3b [11]. This suggestion has not entered guidelines so far.

Most studies evaluating the frequency of malignancy of B3 lesions in excision histology focus on screening-detected mammographic lesions [12–14]. The influence of presentation of the B3 lesion in core biopsies (screen-detected vs. symptomatic) under ultrasound or stereotactic guidance has also been investigated [15]. In a study of 31 patients with high-risk lesions on breast MRI no significant differences considering patient age, indications for breast MRI, size of lesion or morphological features of biopsied lesions was found [16]. The authors concluded that all high-risk lesions diagnosed at MR-guided vacuum-assisted biopsy require surgical excision. Other authors state that in selected cases, if the suspicious lesion is not associated with epithelial atypia, removal by vacuum-assisted biopsy is a safe alternative to surgical excision [13]. Overall, data concerning the outcome of those lesions is still limited and there is no uniform suggestion of further treatment.

Regarding certain B3 lesions, our findings confirm that operative resection is necessary: Particularly ADH show a malignancy rate of 50%. This result is in agreement with a reported malignancy rate of 32 to 59% in mammographic screening [11, 17–19]. The final diagnosis of ADH cannot be made in minimal-invasive biopsy, because here the determination of extensiveness of the lesion is not possible, which is however one of the three main criteria defining ADH [19, 20]. The European Working Group for Breast Screening Pathology therefore recommends to use the term “atypical epithelial proliferation of ductal type” (AEPDT) instead of ADH in diagnostics of minimal-invasive biopsy [2]. The dimension of the lesion is at the same time the most important criterion in the differentiation to low-grade DCIS. Accordingly, the operative resection of an ADH/ AEPDT lesion cannot be questioned.

FEA and LIN also show relevant malignancy rates of 33% respectively 29% in our study and therefore require surgical excision. Crystal et al. observed even higher malignancy rates of 50% for LIN (4/8) and also 50% for FEA (1/2) [14]. Regarding FEA, the precursor lesion of ADH in the low-grade pathway, only very few clinical outcome-studies exist. In observational studies, an association of FEA with lobular neoplasia, ADH, low-grade DCIS, invasive G1 carcinoma and tubular carcinoma is indicated [21]. In approximately one third of subsequent resections, an additional lesion of the low-grade pathway is found [17, 19, 22]. Though, interpretation of these studies is limited because of lacking radiologic-pathologic correlation in several cases and because of different indications for surgical excision. With a malignancy rate of 33% in FEA lesions, our study supports the findings of these previous studies.

Concerning LIN there is still no guideline for the handling of these lesions. Weigel S et al. suggest diagnostic excision of LN lesions only if the lesion is not a coincidental finding accompanying a focal lesion or microcalcifications and if there is a residual lesion detectable after biopsy – with certain radiologic-pathologic correlation [19, 23]. Otherwise annual control mammography is recommended. Our data, however, with a malignancy rate of 29% for LIN, indicates that operative resection in these cases should not be neglected. Therefore especially regarding LIN, further clarification by means of higher patient numbers is required.

Although some studies observed no malignant final histology after MR-VAB of papillary lesions [16] the malignancy rate of our study of 11% for PL however indicates a quite considerable risk for this entity.

In radial scar complexes a more conservative position regarding surgical excision seems justifiable. In our study, out of 6 radial scars, no malignant outcome occurred, which is in accordance with the study of Crystal et al. [16]. Contrarily, Heller S L et al. published data showing high upgrade rates for radial scar lesions at MRI-guided breast biopsies [24]. Hence, regarding this entity, further studies with larger patient cohorts are also essential.

Regarding imaging features predictive of malignancy some authors observed no significant imaging features of upgrade [24]. Here, our study yields some interesting results: mass lesions seem to have a significantly higher malignancy rate than non-mass lesions (31% compared to 0%). This is in accordance with a large retrospective multi-institutional study showing that upgrades of ADH and DCIS diagnosed at MR-guided VAB are significantly associated with the presence of a mass on MR imaging [25]. Furthermore, our study indicates a higher malignant potential for smaller lesions ≤10 mm (PPV 35%) compared to lesions measuring 11–20 mm (PPV 23%) in case of MR-only lesions. Lesions > 20 mm were benign in 100% (10/10). The additional finding that non-mass enhancement in most cases appears as a taller lesion underlines these results. Further investigations in larger patient cohorts are needed to clarify this subject.

With a malignancy rate of 50% for patients with a recently diagnosed carcinoma of the ipsilateral breast, our study confirms the results of Heller et al. stating a significantly higher risk of an upgrade of high-risk lesions in case of ipsilateral cancer or ipsilateral high-risk lesion [24]. Our results also indicate the relevance of previous carcinoma on risk assessment, whereas here our case numbers are too small to be able to differentiate between history of carcinoma of the ipsi- and contralateral breast.

Considering patient age, our data provide no statistical significance, but give clear indication that the outcome of B3 lesions is most unfavorable in the age cohort from 46 to 50 years.

The study has several limitations. Most important, the limited number of patients with 43 inclosed B3 lesions permits only restricted conclusions concerning significance, especially when divided into subgroups. Second, the retrospective study design does not permit to fully reproduce the reasons for indication of surgical excision in all cases. Third, 72% of the patients were referred for breast MRI due to newly diagnosed ipsilateral breast cancer (65%) or history of contraleteral breast cancer (7%), which influences the PPV for malignancy in final histology.

Summarised, the appropriate management of B3 lesions is still discussed controversially in literature. Our study shows that B3 lesions are a summary of different entities with different malignancy rates and that treatment accordingly should be adapted. Further studies with larger patient cohorts and metaanalyses of existing surveys are necessary.

## Conclusions

Our study indicates that management of B3 MR-only lesions of the breast should be adapted to the respective histology. ADH, FEA and LIN should undergo surgical treatment, whereas our data did not show any malignancy in complex sclerosing lesions, non-mass lesions or lesions larger than 2 cm.
